# Obturator Hernia: A Rare Case of Acute Mechanical Intestinal Obstruction

**DOI:** 10.1155/2013/256062

**Published:** 2013-05-02

**Authors:** Ibrahim Aydin, Ahmet Fikret Yucel, Ahmet Pergel, Dursun Ali Sahin

**Affiliations:** Department of Surgery, School of Medicine, Recep Tayyip Erdogan University, 53100 Rize, Turkey

## Abstract

Obturator hernia is a rare type of pelvic hernia which generally occurs in elderly patients with accompanying diseases. Because it is difficult to diagnose before surgery, the morbidity and mortality rates for obturator hernia are high. The most common symptom is strangulation combined with mechanical intestinal obstruction.

## 1. Introduction

Obturator hernia is a type of pelvic hernia in which a bowel segment protrudes through the obturator foramen adjacent to the obturator vessels and nerve. It occurs more frequently in patients with ascites, chronic constipation, and chronic obstructive pulmonary disease and in thin, elderly multiparous women [1]. The most common clinical symptom is strangulation combined with mechanical intestinal obstruction. Because the symptoms are nonspecific, obturator hernia is difficult to diagnose, and most patients are diagnosed during surgery. Obturator hernias occur frequently in elderly patients with accompanying diseases, and therefore the morbidity and mortality rates are high [2]. Currently, diagnostic imaging, especially computed tomography, is widely used to diagnose obturator hernias before surgery in the early stages of the disease [3]. The aim of this report was to present the case, accompanied by relevant literature, of a patient with mechanical intestinal obstruction who was diagnosed with obturator hernia using computed tomography. 

## 2. Case Report

**Figure 1:**
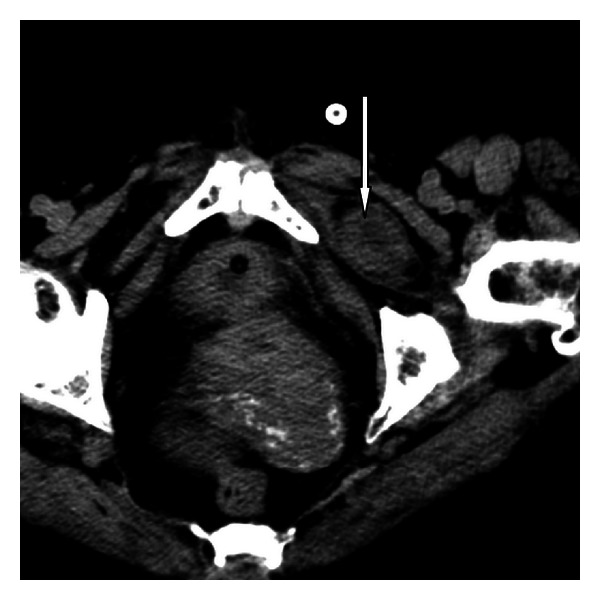
Computed tomography image showing a herniated bowel loop in the obturator foramen on the left.

**Figure 2:**
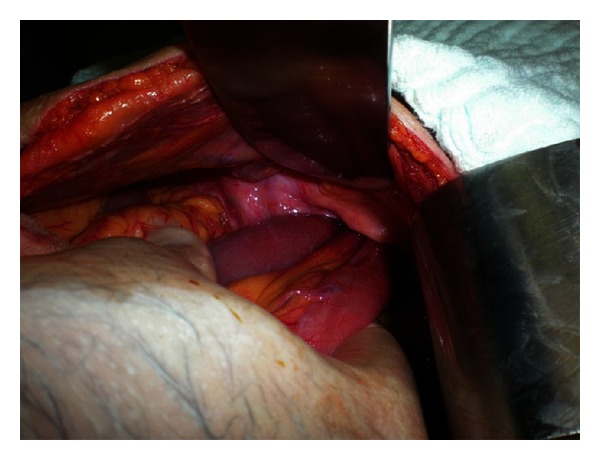
Strangulated small intestine segment protruding through the obturator canal.

**Figure 3:**
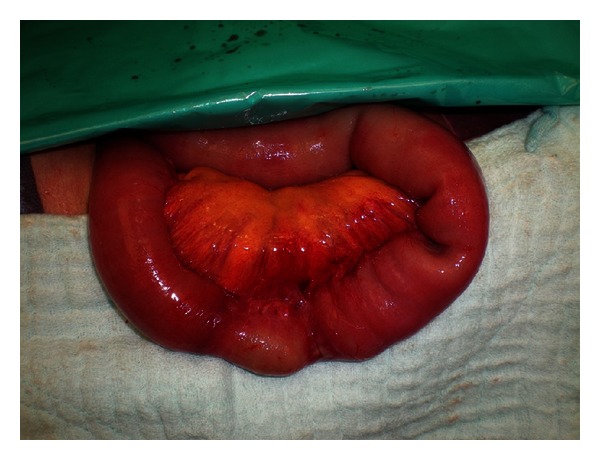
Strangulated small intestinal segment.

A 76-year-old female patient weighing 42 kg presented to the ER with a four-day history of abdominal pain, abdominal swelling, nausea, vomiting, constipation, and inability to pass gas. Her physical examination revealed abdominal distention and tenderness, and she had hyperactive bowel sounds. No palpable hernia was located. Her lab tests were normal except for elevated white blood cell count (12500/mm³). Direct abdominal X-ray images obtained in a standing position revealed dilated loops in the small intestines and gas fluid levels. Her computed tomography results showed a small intestine loop completely herniated through the obturator foramen and dilatation in the proximal small bowel (Figure 1). The patient was diagnosed with obturator hernia and underwent surgery. During surgery, a strangulated small intestine segment extending through the left obturator foramen was detected (Figure 2). After reduction, the obturator foramen was closed with primary sutures (Figure 3). The patient did not experience any postoperative problems and was discharged on the fifth day after admittance. 

## 3. Discussion

Obturator hernia was first described by Armaud de Ronsil in 1724 and was successfully treated for the first time by Henry Obre in 1851 [4, 5]. Obturator hernia protrudes through the circle surrounded by the superior ramus of the pelvic bone in the front, the obturator membrane and the internal and external obturator muscles on the inferior side, and the obturator vessels and the nerves on the posterolateral aspect. Obturator hernias account for 0.05–0.4% of all abdominal hernias [6]. It is often referred to as “little old lady's hernia” [5], and our case was also an elderly female patient with a low body mass index. 

Due to its nonspecific symptoms, obturator hernia is difficult to diagnose. Most patients undergo surgery because of an intestinal obstruction and are diagnosed during the operation. The preoperative diagnosis rate is reported as only 10–30% [2]. More than 90% of patients with obturator hernia are admitted to the hospital with acute intestinal obstruction, presenting with abdominal pain, nausea, and vomiting [7]. The Howship-Romberg sign pain along the medial aspect of the thigh to the knee due to compression of the anterior branch of the obturator nerve by the contents of the hernia is present in 50% of patients [8, 9]. However, the sign is commonly mistaken for neuromuscular pain, as joint pain is common in elderly patients and is overlooked. Another clinical sign of obturator hernia is the Hannington-Kiff sign, in which the adductor reflex is absent in the thigh.

Our case was an elderly multiparous patient with a low body, mass index and negative Howship-Romberg sign. Clinically diagnosing obturator hernia is difficult because the symptoms are nonspecific. A preoperative diagnosis can be determined during diagnostic imaging methods, such as ultrasonography and computed tomography. Of these methods, computed tomography has high sensitivity and specificity [3]. Our case was diagnosed using computed tomography and treated in the early stages of the disease.

In conclusion, obturator hernia must be considered in the differential diagnosis of thin, elderly patients, especially females, admitted with symptoms of intestinal obstruction. Computed tomography must be used for diagnosis because of its high sensitivity rate. 

## References

[1] Ziegler DW, Rhoads JE (1995). Obturator hernia needs a laparotomy, not a diagnosis. *American Journal of Surgery*.

[2] Gray SW, Skandalakis JE, Soria RE, Rowe JS (1974). Strangulated obturator hernia. *Surgery*.

[3] Ijiri R, Kanamaru H, Yokoyama H, Shirakawa M, Hashimoto H, Yoshino G (1996). Obturator hernia: the usefulness of computed tomography in diagnosis. *Surgery*.

[4] Hsu CH, Wang CC, Jeng LB, Chen MF (1988). Obturator hernia. *Surgery, Gynecology & Obstetrics*.

[5] Dundamadappa SK, Tsou IYY, Goh JSK (2006). Clinics in diagnostic imaging (107). *Singapore Medical Journal*.

[6] Chang SS, Shan YS, Lin YJ, Tai YS, Lin PW (2005). A review of obturator hernia and a proposed algorithm for its diagnosis and treatment. *World Journal of Surgery*.

[7] Lo CY, Lorentz TG, Lau PWK (1994). Obturator hernia presenting as small bowel obstruction. *American Journal of Surgery*.

[8] Kammori M, Mafune KI, Hirashima T (2004). Forty-three cases of obturator hernia. *American Journal of Surgery*.

[9] Sorabella RA, Miniati DN, Brandt ML (2005). Laparoscopic obturator hernia repair in an adolescent. *Journal of Pediatric Surgery*.

